# Trans-ethnic meta-regression of genome-wide association studies accounting for ancestry increases power for discovery and improves fine-mapping resolution

**DOI:** 10.1093/hmg/ddx280

**Published:** 2017-07-17

**Authors:** Reedik Mägi, Momoko Horikoshi, Tamar Sofer, Anubha Mahajan, Hidetoshi Kitajima, Nora Franceschini, Mark I. McCarthy, COGENT-Kidney Consortium, T2D-GENES Consortium, Andrew P. Morris

**Affiliations:** 1 Estonian Genome Center, University of Tartu, Tartu, Estonia; 2 Wellcome Trust Centre for Human Genetics, University of Oxford, Oxford, UK; 3 Laboratory for Endocrinology, Metabolism and Kidney Diseases, RIKEN, Center for Integrative Medical Sciences, Yokohama, Japan; 4 Department of Biostatistics, University of Washington, Seattle, WA, USA; 5 Department of Epidemiology, University of North Carolina, Chapel Hill, NC, USA; 6 Oxford Centre for Diabetes, Endocrinology and Metabolism, Radcliffe Department of Medicine, University of Oxford, Oxford, UK; 7 Oxford NIHR Biomedical Research Centre, Oxford University Hospitals Trust, Oxford, UK; 8 Department of Biostatistics; 9 Department of Molecular and Clinical Pharmacology, University of Liverpool, Liverpool, UK

## Abstract

Trans-ethnic meta-analysis of genome-wide association studies (GWAS) across diverse populations can increase power to detect complex trait loci when the underlying causal variants are shared between ancestry groups. However, heterogeneity in allelic effects between GWAS at these loci can occur that is correlated with ancestry. Here, a novel approach is presented to detect SNP association and quantify the extent of heterogeneity in allelic effects that is correlated with ancestry. We employ trans-ethnic meta-regression to model allelic effects as a function of axes of genetic variation, derived from a matrix of mean pairwise allele frequency differences between GWAS, and implemented in the MR-MEGA software. Through detailed simulations, we demonstrate increased power to detect association for MR-MEGA over fixed- and random-effects meta-analysis across a range of scenarios of heterogeneity in allelic effects between ethnic groups. We also demonstrate improved fine-mapping resolution, in loci containing a single causal variant, compared to these meta-analysis approaches and PAINTOR, and equivalent performance to MANTRA at reduced computational cost. Application of MR-MEGA to trans-ethnic GWAS of kidney function in 71,461 individuals indicates stronger signals of association than fixed-effects meta-analysis when heterogeneity in allelic effects is correlated with ancestry. Application of MR-MEGA to fine-mapping four type 2 diabetes susceptibility loci in 22,086 cases and 42,539 controls highlights: (i) strong evidence for heterogeneity in allelic effects that is correlated with ancestry only at the index SNP for the association signal at the *CDKAL1* locus; and (ii) 99% credible sets with six or fewer variants for five distinct association signals.

## Introduction

There is increasing evidence from genome-wide association studies (GWAS) that common SNPs driving complex human trait associations are shared across diverse populations (1,2), and furthermore, that alleles at these signals demonstrate concordant directions of effect across ethnicities (3). This observation is consistent with a model in which causal variants are shared across diverse populations, for which trans-ethnic meta-analysis offers an opportunity to increase power to detect novel loci through increased sample size. However, heterogeneity in allelic effects between GWAS at SNPs in these loci, which cannot be accommodated through traditional fixed-effects meta-analysis, but which is correlated with ancestry, can occur for several reasons. First, variability in patterns of linkage disequilibrium (LD) with the causal variant(s) between ethnic groups will propagate heterogeneity between populations in the allelic effects of SNPs, which has the advantage of enabling high-resolution fine-mapping (4–6). Second, the causal variant(s) may interact with an environmental risk factor that differs in exposure across populations, or with SNPs that differ in allele frequency between ethnic groups, thereby generating heterogeneity in allelic main effects unless accounted for in the analysis. Third, the quality of imputation might vary between populations, dependent on the reference panel used, leading to downward bias in allelic effect estimates within ethnic groups in which genotypes are less well predicted.

One approach to allow for heterogeneity in allelic effects between GWAS is to utilise meta-analysis under a random-effects model. The RE2 meta-analysis increases power over the traditional random-effects model by taking account of the expected homogeneity of allelic effects between GWAS under the null hypothesis of no association for which all allelic effects are zero (7). However, these models do not assume any structure to the heterogeneity in allelic effects between populations that would be expected in trans-ethnic meta-analysis. To account for this structure, MANTRA implements a Bayesian partition model that clusters GWAS using a prior model of similarity between them, assessed by mean pairwise genome-wide allele frequency differences (8). Compared to fixed- and random-effects meta-analysis, MANTRA has been demonstrated to increase power to detect association and improve the resolution of trans-ethnic fine-mapping across a range of heterogeneity scenarios (8,9). Nevertheless, MANTRA utilises Markov chain Monte Carlo methods to approximate the posterior distribution of model parameters, which can be computationally intractable for meta-analysis of large numbers of GWAS and SNPs. For trans-ethnic fine-mapping, methodology integrating association summary statistics and functional annotation to improve localisation of causal variants has been implemented in PAINTOR (10), although this approach does not take account of the genetic similarity between GWAS to inform the structure of heterogeneity in allelic effects.

To address the shortcomings of existing methodologies for aggregating GWAS from diverse populations, we have developed a novel approach to detect and fine-map complex trait association signals via trans-ethnic meta-regression. This approach uses genome-wide metrics of diversity between populations to derive axes of genetic variation via multi-dimensional scaling. Allelic effects of a variant across GWAS, weighted by their corresponding standard errors, can then be modelled in a linear regression framework, including the axes of genetic variation as covariates. The flexibility of this model enables partitioning of the heterogeneity into components that are correlated with ancestry and residual variation, which would be expected to improve fine-mapping resolution. Here, we present the results of a detailed simulation study to investigate the properties of trans-ethnic meta-regression for the detection and fine-mapping of loci containing a single causal variant contributing to a binary phenotype over a range of scenarios for heterogeneity in allelic effects between diverse populations. We compare the performance of the meta-regression with fixed- and random-effects (RE2) meta-analysis, implemented in METASOFT (7), and with MANTRA (8) and PAINTOR (10) in the context of fine-mapping. We also present the results of an application of trans-ethnic meta-regression to: (i) GWAS of kidney function in 71,461 individuals of African American, East Asian, European and Hispanic/Latino ancestry from the COGENT-Kidney Consortium (11) and; (ii) fine-mapping four type 2 diabetes (T2D) susceptibility loci in 22,086 cases and 42,539 controls of East Asian, European, South Asian, African American and Mexican American ancestry from the T2D-GENES Consortium (12).

## Results

We have developed a novel approach to aggregate association summary statistics across GWAS from diverse populations to account for heterogeneity in allelic effects that is correlated with ancestry (Materials and Methods). Briefly, we employ trans-ethnic meta-regression to model allelic effects as a function of axes of genetic variation, derived from a matrix of mean pairwise allele frequency differences between GWAS. The meta-regression model partitions heterogeneity in allelic effects between GWAS into two components: (i) heterogeneity that is correlated with ancestry; and (ii) residual heterogeneity. Bayes’ factors in favour of association can be derived from the meta-regression model for each variant, enabling fine-mapping and construction of credible sets. The meta-regression methodology has been implemented in the MR-MEGA (Meta-Regression of Multi-Ethnic Genetic Association) software (http://www.geenivaramu.ee/en/tools/mr-mega).

### Simulation study design

We began by undertaking a detailed simulation study to compare the performance of the meta-regression methodology with existing approaches for discovery and fine-mapping of GWAS loci across diverse populations. We considered the 26 reference populations from Phase 3 of the 1000 Genomes Project (13), incorporating haplotypes of African, East Asian, European, Native American and South Asian ancestry (Supplementary Material, Table S1). We used a subset of 13,189 autosomal variants from the reference panel with minor allele frequency (MAF) > 5% in all populations, and separated by at least 1 Mb, to derive the matrix of pairwise Euclidean distances between the populations. We then implemented multi-dimensional scaling of the distance matrix to derive three axes of genetic variation to separate populations between ancestry groups (Supplementary Material, Fig. S1).

We considered a range of models of association of a causal variant with a binary phenotype across ancestry groups, parameterised in terms of the allelic effect (odds-ratio, *ψ*) in each population (Supplementary Material, Table S1). These scenarios incorporated heterogeneity in allelic effects of the causal variant between ancestry groups: (i) homogenous; (ii) African-specific; (iii) Eurasian; (iv) Native American; (v) random (non-ancestral). Under model (i), the allelic effect of the causal variant is homogeneous across all populations. Under model (ii), the allelic effect of the causal variant is specific to populations of African ancestry. Under model (iii), the allelic effect of the causal variant is zero in populations of African ancestry, and heterogeneous between populations of East Asian ancestry and those of European, South Asian and Native American ancestry. Under model (iv), the allelic effect of the causal variant is specific to, but heterogeneous between, populations of Native American ancestry. Finally, under model (v), the allelic effect of the causal variant is specific to one population in each ancestry group.

### Simulation study: false positive error rate and power

To assess false positive error rates and power for each scenario, we generated 1,000 replicates of genotype data for the causal variant in 1,000 cases and 1,000 controls from each population (Materials and Methods). Association summary statistics for the causal variant were aggregated across populations using the meta-regression model, implemented in MR-MEGA, including three axes of genetic variation as covariates to separate ancestry groups. For comparison, we also aggregated association summary statistics via fixed-effects (inverse-variance weighted log-odds ratios) and random-effects (RE2) meta-analysis implemented in METASOFT (7). We have not included MANTRA in our comparisons of methods for false positive error rates and power because: (i) the increased computational burden makes simulations intractable and; (ii) the required derivation of nominal and genome-wide significance thresholds for Bayes’ factors in favour of association across the allele frequency spectrum is not straightforward.

False positive error rates for detecting association were consistent with the nominal significance threshold (*P *<* *0.05), across all heterogeneity scenarios considered, for fixed- and random-effects meta-analysis, and for meta-regression including three axes of genetic variation to account for heterogeneity in allelic effects between ancestry groups (Supplementary Material, Table S2).

**Figure 1.**
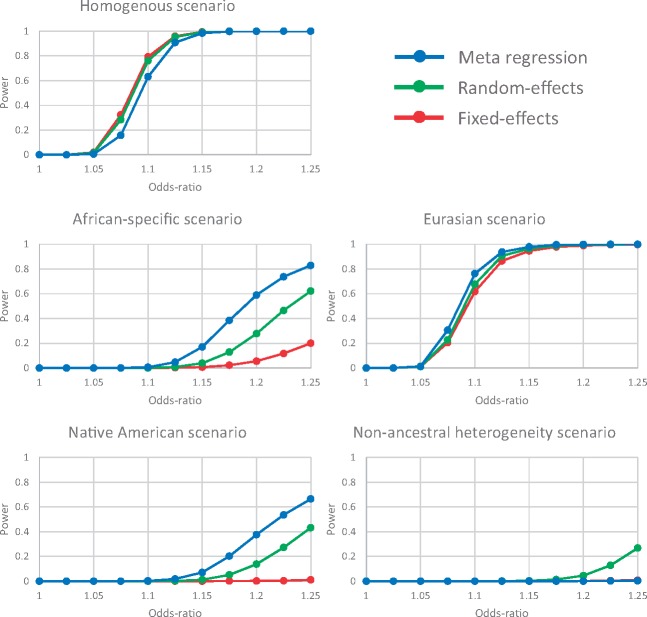
Power to detect association, at genome-wide significance (*P *<* *5 × 10^−8^), using alternative approaches to aggregate GWAS across diverse populations: fixed-effects meta-analysis; random-effects (RE2) meta-analysis; and meta-regression including axes of genetic variation as covariates as implemented in MR-MEGA. Power is presented as a function of the allelic odds-ratio for each of five scenarios for heterogeneity in effects between populations, described in Supplementary Material, **Table S1.**

For scenarios in which heterogeneity in allelic effects between populations was correlated with ancestry (African-specific, Eurasian and Native American), greatest power to detect association was attained for the meta-regression including three axes of genetic variation as covariates (Fig. 1). The gains in power over fixed- and random-effects meta-analysis were greatest when the effect of the variant was specific to one ancestry group (African-specific and Native American). For all three of these scenarios, power to detect heterogeneity in allelic effects that is correlated with ancestry in the meta-regression model is greater than that obtained from Cochran’s *Q* statistic in the fixed-effects meta-analysis (Supplementary Material, Fig. S2).

For the scenario in which heterogeneity in allelic effects between populations is random (non-ancestral), power was low for all methods, but greatest for random-effects meta-analysis (Fig. 1). As expected, power to detect heterogeneity in allelic effects that is correlated with ancestry in the meta regression model attained the nominal significance threshold (*P *<* *0.05) for this scenario (Supplementary Material, Fig. S2). Power to detect residual heterogeneity in allelic effects in the meta-regression model or via Cochran’s *Q* statistic in the fixed-effects meta-analysis was equivalent.

Finally, for the scenario of homogenous allelic effects across populations, greatest power to detect association was attained through fixed-effects meta-analysis, as expected (Fig. 1). There was only a small reduction in power for random-effects (RE2) meta-analysis, which appropriately accounts for the lack of heterogeneity under the null hypothesis of no association (7). There was a further small reduction in power for the meta-regression model, which was penalised for the additional parameters required for the axes of genetic variation that do not contribute to heterogeneity in allelic effects between populations in this scenario. For this scenario, power to detect heterogeneity in allelic effects that is correlated with ancestry in the meta regression model attained the nominal significance threshold (*P *<* *0.05), as expected (Supplementary Material, Fig. S2). Power to detect residual heterogeneity in allelic effects in the meta-regression model or via Cochran’s *Q* statistic in the fixed-effects meta-analysis also attained the nominal significance threshold.

### Simulation study: fine-mapping loci with a single causal variant

To assess fine-mapping resolution within loci containing a single causal variant, for each scenario, we generated 500 replicates of genotype data for variation in a 2 Mb genomic region, in 1,000 cases and 1,000 controls for each population (Materials and Methods). For each replicate, we considered two settings: (i) ‘perfect’ data, where all variants in the region were captured, with no missing genotypes or errors, for benchmarking purposes; and (ii) ‘imperfect’ data, where only 100 randomly selected variants in the 2 Mb region were retained, to represent a typical GWAS array, and the resulting scaffold of genotypes was imputed up to haplotypes from the 1000 Genomes Project Phase 3 reference panel (13) (Material and Methods). For each replicate, for both ‘perfect’ and ‘imperfect’ data settings, we obtained the posterior probability of driving the association for each variant from the meta-regression model, implemented in MR-MEGA, including three axes of genetic variation as covariates to separate ancestry groups. For comparison, posterior probabilities of driving the association were derived, for each variant, from: (i) fixed- and random-effects meta-analysis, implemented in METASOFT (7); (ii) MANTRA (8); and (iii) PAINTOR (10), assuming a single causal variant at the locus and approximating LD between variants in each population from haplotypes in the 1000 Genomes Project Phase 3 reference panel (13). Note that we did not run PAINTOR in a mode to infer functional enrichment because our simulations did not use annotation to weight the selection of the causal variant in the region. In each replicate, we used posterior probabilities from each of the five methods to construct the 99% credible set driving the association signal at the locus (Materials and Methods).

We considered three metrics of fine-mapping performance across simulations: (i) the number of variants in the 99% credible set; (ii) the mean posterior probability ascribed to the causal variant; and (iii) the coverage of the causal variant by the 99% credible set. Smaller credible sets correspond to fine-mapping at higher resolution, whilst the mean posterior probability for the causal variant measures accuracy. For each heterogeneity scenario, we considered population-specific odds-ratios with approximately 80% power to detect association with the meta-regression model (Fig. 1): homogeneous, *ψ *= 1.10; African-specific, *ψ *= 1.25; Eurasian, *ψ *= 1.10; Native American, *ψ *= 1.30; and non-ancestral, *ψ *= 1.35.

**Figure 2.**
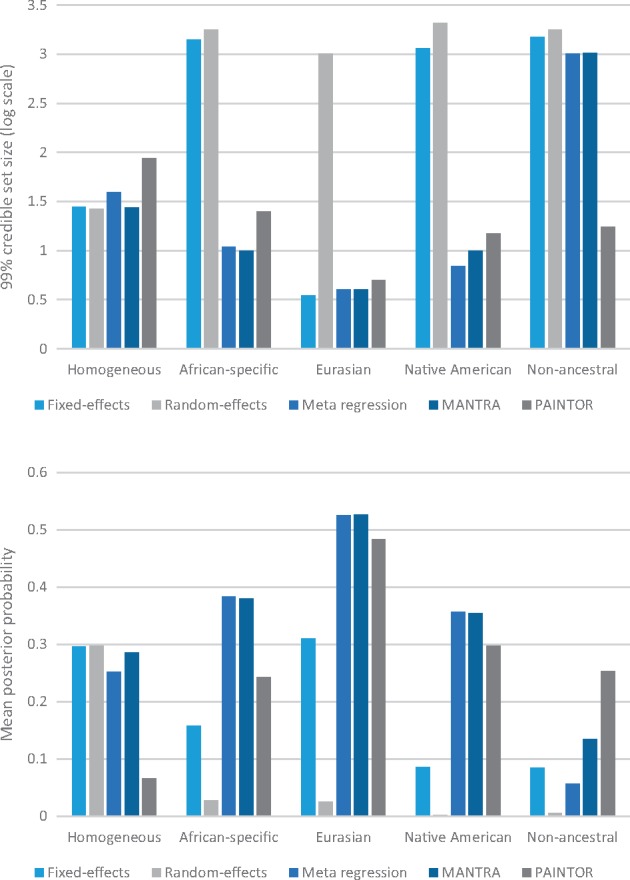
Metrics of fine-mapping resolution, with ‘perfect data’, across alternative approaches to aggregate GWAS across diverse populations: fixed-effects meta-analysis; random-effects meta-analysis; meta-regression including axes of genetic variation as covariates as implemented in MR-MEGA; MANTRA; and PAINTOR. Two metrics are presented: (i) the median number of variants in the 99% credible set on a log_10_-scale; and (ii) the mean posterior probability ascribed to the causal variant. Metrics are presented for each of five scenarios for heterogeneity in effects between populations, described in Supplementary Material, Table S1. In each scenario, the odds ratio has been fixed to obtain approximately 80% power to detect association at genome-wide significance (*P *<* *5 × 10^−8^) in the meta-regression analysis.

We first considered the ‘perfect’ data setting, where all variants in the region were captured, with no missing genotypes or errors (Fig. 2, Table 1). First, we note that, across the range of scenarios considered, the coverage of the causal variant by the credible set obtained from PAINTOR was not consistent with 99%, suggesting that this method is not well calibrated in our simulations. Only meta-regression, as implemented in MR-MEGA, attained coverage rates for the causal variant that were consistent with 99% across all heterogeneity scenarios. For scenarios in which heterogeneity in allelic effects was correlated with ancestry (African-specific, Eurasian and Native American), the resolution and accuracy of fine-mapping was always substantially worse for the fixed- or random-effects meta-analysis, with the meta-regression model and MANTRA performing better than PAINTOR. For example, for the Native American scenario, the median number of SNPs in the 99% credible set was 1,156 and 2,063 for fixed- and random-effects, respectively, whilst for the meta-regression, MANTRA and PAINTOR was just 7, 10 and 15, respectively. This improved fine-mapping resolution reflects the increased power obtained through modelling of heterogeneity in allelic effects between GWAS that is correlated with ancestry. For the scenario in which heterogeneity in allelic effects between populations is random (non-ancestral), PAINTOR outperformed all other methods in terms of fine-mapping resolution and accuracy. For this scenario, axes of genetic variation that distinguish broad ethnic groups in the meta-regression model cannot fully account for non-ancestral heterogeneity between GWAS. Finally, for the scenario of homogenous allelic effects across populations, the number of variants in the 99% credible set was similar across the range of meta-analysis methods considered. However, the mean posterior probability for the causal variant was substantially lower for PAINTOR than the other fine-mapping methods.

**Table 1.** Coverage of the causal variant by the 99% credible set across 500 simulations of each scenario with ‘perfect’ data for five fine-mapping approaches: (i) fixed-effects meta-analysis; (ii) random-effects meta-analysis; (iii) meta-regression accounting for heterogeneity in allelic effects implemented in MR-MEGA; (iv) MANTRA; and (v) PAINTOR

Fine-mapping method	Heterogeneity scenario				
	Homogeneous	African-specific	Eurasian	Native American	Non-ancestral
Fixed-effects	**0.998**	**0.986**	0.660	0.944	**0.996**
Random-effects	**1.000**	**0.990**	0.966	**1.000**	**0.998**
Meta-regression	**0.992**	**0.998**	**0.978**	**0.992**	**0.980**
MANTRA	**0.994**	**0.982**	**0.992**	0.918	0.878
PAINTOR	0.692	0.772	0.972	0.916	0.922

Coverage rates highlighted in bold are consistent with 99% (based on 500 simulations of each of the five scenarios).

**Figure 3.**
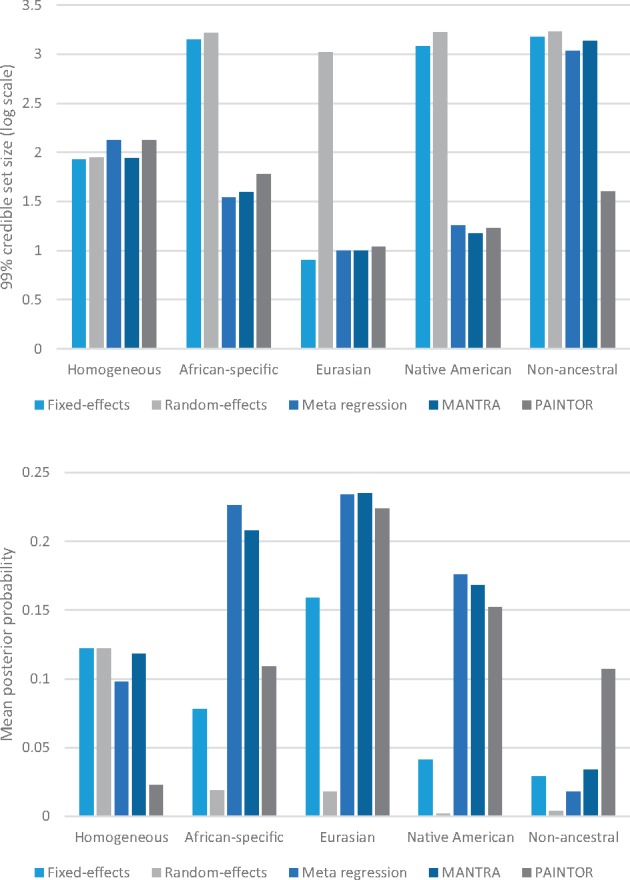
Metrics of fine-mapping resolution, with imputed data, across alternative approaches to aggregate GWAS across diverse populations: fixed-effects meta-analysis; random-effects meta-analysis; meta-regression including axes of genetic variation as covariates as implemented in MR-MEGA; MANTRA; and PAINTOR. Two metrics are presented: (i) the median number of SNPs in the 99% credible set on a log_10_-scale; and (ii) the mean posterior probability ascribed to the causal variant. Metrics are presented for each of five scenarios for heterogeneity in effects between populations, described in Supplementary Material, Table S1. In each scenario, the odds ratio has been fixed to obtain approximately 80% power to detect association at genome-wide significance (*P *<* *5 × 10^−8^) in the meta-regression analysis.

We then considered the more realistic ‘imperfect’ data setting, in which a subset of genetic variation across a locus was assayed directly with a GWAS array, with subsequent imputation up to haplotypes from the 1000 Genomes Project Phase 3 reference panel (13). Coverage of the causal variant by the 99% credible set was reduced for all methods across the range of scenarios considered (Supplementary Material, Table S3). This reduced coverage reflects that the causal variant may not always be well imputed across all populations, and thus may have reduced association signal compared with other variants at the locus, resulting in exclusion from the credible set. The relative performance of the methods with imputed data across the range of scenarios considered was consistent with that observed for ‘perfect data’, although the posterior probability ascribed to the causal variant was lower (Fig. 3).

We also compared, across simulations, the computational burden of each of the trans-ethnic meta-analysis approaches to assess association with variants within the locus (Supplementary Material, Table S4). Using a dedicated single core processor, MANTRA was the most computationally expensive (mean run time of 66 minutes), compared to less than two minutes for all other methods.

### Trans-ethnic meta-analysis of GWAS of kidney function

We considered nine GWAS of kidney function, assessed by the estimated glomerular filtration rate (eGFR), in 71,461 individuals of African American, East Asian, European and Hispanic/Latino ancestry (Supplementary Material, Table S5). Analyses of these GWAS, including 71,638 individuals, have been previously reported by the COGENT-Kidney Consortium (11). However, since publication of these results, 177 individuals from HCHS/SOL have withdrawn consent, and association analyses have been repeated for this cohort. Each GWAS was imputed up to the 1000 Genomes Project Phase 1 reference panel (14), and each variant passing quality control was tested for association with eGFR (Materials and Methods). Association summary statistics for each variant were aggregated across studies via: (i) fixed-effects meta-analysis, implemented in METASOFT (7); and (ii) trans-ethnic meta-regression, implemented in MR-MEGA, including the two axes of genetic variation as covariates (Materials and Methods, Supplementary Material, Fig. S3).

Genome-wide, we observed strong correlation in association *P*-values for eGFR from the trans-ethnic meta-regression and the fixed-effects meta-analysis (Supplementary Material, Fig. S4). Stronger signals of association with eGFR were observed from the meta-regression when there was heterogeneity in allelic effects between GWAS that was correlated with ancestry. A total of 16 loci attained genome-wide significant evidence (*P *<* *5×10^−8^) of association with eGFR from the trans-ethnic meta-regression (Table 2), with the strongest signals observed at/near *SLC34A1* (rs35716097, *P *=* *3.0×10^−17^), *SHROOM3* (rs28394165, *P *=* *1.8×10^−15^), *UNCX* (rs62435145, *P *=* *8.3×10^−15^) and *PDILT-UMOD* (rs77924615, *P *=* *9.7×10^−15^). Signals of association at these loci were stronger from the fixed-effects meta-analysis than the meta-regression when the lead SNP demonstrated little evidence of heterogeneity in allelic effects between GWAS. The strongest evidence of heterogeneity in allelic effects in the fixed effects meta-analysis, as assessed by Cochran’s *Q* statistic, was observed for the lead SNP at *WDR72* (rs690428, *P *=* *7.8×10^−5^). In the meta-regression analysis, the heterogeneity was partially correlated with ancestry (*P *=* *0.00053), where allelic effects of the lead SNP on eGFR are specific to populations of European and East Asian descent (Supplementary Material, Fig. S5).

**Table 2.** Loci attaining genome-wide significant evidence of association (*P *<* *5 × 10^−8^) with eGFR in MR-MEGA meta-regression of 71,461 individuals

Locus	Lead SNP	Chr	Position(bp, b37)	Alleles		Fixed-effects meta-analysis				MR-MEGA meta-regression		
				Effect	Other	Beta	SE	*P*-value	*p_Q_*	*P*-value	*p*_HET-ANC_	*p*_HET-RES_
*SLC43A1*	rs35716097	5	176,806,636	T	C	−1.092	0.128	3.5 × 10^−17^	0.13	3.0 × 10^−17^	0.016	0.66
*SHROOM3*	rs28394165	4	77,394,018	C	T	−0.949	0.117	1.0 × 10^−15^	0.0028	1.8 × 10^−15^	0.041	0.036
*UNCX*	rs62435145	7	1,286,567	T	G	−1.097	0.138	4.0 × 10^−15^	0.16	8.3 × 10^−15^	0.042	0.58
*PDILT-UMOD*	rs77924615	16	20,392,332	G	A	−1.184	0.147	1.9 × 10^−15^	0.010	9.7 × 10^−15^	0.10	0.017
*BCAS3*	rs9895661	17	59,456,589	C	T	−0.990	0.132	1.7 × 10^−13^	0.18	6.5 × 10^−13^	0.085	0.38
*GCKR*	rs1260326	2	27,730,940	C	T	−0.867	0.115	9.0 × 10^−14^	0.072	2.0 × 10^−12^	0.54	0.041
*WDR72*	rs690428	15	53,950,578	A	C	−0.688	0.115	4.1 × 10^−9^	7.8 × 10^−5^	8.7 × 10^−11^	0.00053	0.0081
*CPS1*	rs715	2	211,543,055	C	T	−0.880	0.128	1.1 × 10^−11^	0.21	1.3 × 10^−10^	0.31	0.21
*SPATA5L1-GATM*	rs2486288	15	45,712,339	C	T	−0.875	0.126	7.5 × 10^−12^	0.73	1.8 × 10^−10^	0.65	0.63
*ALMS1*	rs11884776	2	73,746,923	C	T	−0.929	0.141	7.6 × 10^−11^	0.15	3.3 × 10^−10^	0.59	0.035
*LRP2*	rs57989581	2	170,194,459	C	A	−1.961	0.315	8.6 × 10^−10^	0.19	7.7 × 10^−10^	0.025	0.70
*PIP5K1B*	rs4744712	9	71,434,707	A	C	−0.756	0.112	2.8 × 10^−11^	0.90	8.1 × 10^−10^	0.80	0.80
*PRKAG2*	rs10265221	7	151,414,329	C	T	−0.952	0.146	1.2 × 10^−10^	0.24	1.9 × 10^−9^	0.44	0.19
*DAB2-C9*	chr5:39404526:D	5	39,404,526	D	R	−0.822	0.126	1.2 × 10^−10^	0.79	2.2 × 10^−9^	0.56	0.74
*SLC22A2*	rs316009	6	160,675,764	C	T	−1.190	0.193	1.2 × 10^−9^	0.48	1.4 × 10^−8^	0.36	0.49
*LOC100132354-VEGFA*	rs881858	6	43,806,609	A	G	−0.777	0.127	1.6 × 10^−9^	0.0019	2.1 × 10^−8^	0.40	0.00092

Chr: chromosome. SE: standard error. *p_Q_*: Cochran’s *Q P*-value. *p*_HET-ANC_: *P*-value for heterogeneity correlated with ancestry. *p*_HET-RES_: *P*-value for residual heterogeneity.

### Fine-mapping of four T2D susceptibility loci: *CDKAL1*, *CDKN2A-B*, *IGF2BP2* and *KCNQ1*

We considered 18 GWAS of T2D susceptibility in 22,086 T2D cases and 42,539 controls of East Asian, European, South Asian, African American and Mexican American ancestry (Supplementary Material, Table S6), analyses of which have been previously reported by the T2D-GENES Consortium (12). In their study, each GWAS was imputed up to the 1000 Genomes Project Phase 1 reference panel (14) for the four loci, and each variant passing quality control was tested for association with T2D susceptibility. Association summary statistics for each variant were then aggregated across GWAS using MANTRA (8), and step-wise conditional analyses revealed a total of seven distinct signals of association across the four loci, three mapping to *KCNQ1*, two to *CDKN2A-B*, and one each at *IGF2BP2* and *CDKAL1*.

For each distinct association signal, we applied the meta-regression model, implemented in MR-MEGA, including three axes of genetic variation as covariates (Materials and Methods, Supplementary Material, Fig. S6). We observed genome-wide significant evidence of T2D association (*P *<* *5×10^−8^) for index SNPs for each distinct signal across the four susceptibility loci from meta-regression accounting for ancestry with three axes of genetic variation as covariates (Table 3, Supplementary Material, Fig. S7). We observed strong evidence for heterogeneity in allelic effects that is correlated with ancestry only at the index SNP for the association signal at the *CDKAL1* locus (rs9368222, *P *=* *0.00042). The heterogeneity was primarily accounted for by the third axis of genetic variation (*P *=* *0.0046), which separates GWAS of South Asian ancestry from those of African American, East Asian, European and Mexican American descent (Supplementary Material, Fig. S6). Allelic effect sizes increased along this axis (log-odds ratio 2.69, standard error 0.81), suggesting that rs9368222 has weaker effects on T2D susceptibility in South Asian populations (Supplementary Material, Fig. S8). These data are consistent with previous reports of heterogeneity at the *CDKAL1* locus (15,16), where allelic effects are stronger in European and East Asian ancestry populations than in other ethnic groups.

**Table 3.** Index SNPs for distinct T2D association signals at four susceptibility loci on the basis of aggregation of summary statistics from 18 GWAS (22,086 cases and 42,539 controls) from diverse populations using: (i) MR-MEGA meta-regression accounting for ancestry with three axes of genetic variation as covariates; and (ii) reported results from fixed-effects meta-analysis

Locus	Index SNP	Chr	Position	Alleles		MR-MEGA meta-regression			Fixed-effects meta-analysis		
				Risk	Other	*P*-value	*p*_HET-ANC_	*p*_HET-RES_	OR (95% CI)	*P*-value	*p_Q_*
*IGF2BP2*	rs11705729	3	185,507,299	T	C	2.1 × 10^−19^	0.50	0.44	1.14 (1.11–1.17)	1.3 × 10^−21^	0.49
*CDKAL1*	rs9368222	6	20,686,996	A	C	5.1 × 10^−31^	0.00042	0.23	1.17 (1.14–1.21)	4.1 × 10^−30^	0.0058
*CDKN2A-B*	rs10965246	9	22,132,698	T	C	4.8 × 10^−37^	0.62	0.0012	1.31 (1.26–1.36)	8.4 × 10^−40^	0.0029
	rs10757282	9	22,133,984	C	T	3.9 × 10^−11^	0.16	0.24	1.12 (1.09–1.16)	2.0 × 10^−12^	0.17
*KCNQ1*	rs231353	11	2,709,019	G	A	2.7 × 10^−9^	0.92	0.64	1.11 (1.07–1.14)	1.7 × 10^−11^	0.79
	rs233448	11	2,840,424	C	T	3.9 × 10^−10^	0.34	0.17	1.12 (1.09–1.16)	9.5 × 10^−12^	0.18
	rs2237897	11	2,858,546	C	T	2.9 × 10^−10^	0.33	0.36	1.19 (1.14–1.26)	7.7 × 10^−12^	0.35

Chr: chromosome. OR: odds-ratio. CI: confidence interval. *p*_HET-ANC_: *P*-value for heterogeneity correlated with ancestry. *p*_HET-RES_: *P*-value for residual heterogeneity. *p_Q_*: Cochran’s *Q* statistic *P*-value.

Construction of 99% credible sets of variants driving distinct association signals across the four susceptibility loci revealed that the resolution of fine-mapping attained from meta regression was equivalent to that previously reported using MANTRA (12) (Table 4). The most precise localisation was observed for two of the association signals at the *KCNQ1* locus, indexed by rs2237897 (4 variants mapping to 342 bp of an intron of *KCNQ1*) and rs231353 (4 variants mapping to 38.5 kb of *KCNQ1-OT1*). At the *CDKN2A-B* locus, the 99% credible sets for both association signals incorporate a total of 12 non-overlapping variants that map to the same <5 kb interval. Annotation of the 99% credible sets revealed inclusion of no coding variants, consistent with previous reports that T2D association signals at these four loci are most likely to be mediated through gene regulation (12,17).

**Table 4.** Properties of 99% credible sets of variants underlying distinct T2D association signals at four susceptibility loci on the basis of aggregation of association summary statistics from 18 GWAS (22,086 cases and 42,539 controls) from diverse populations using: (i) MR-MEGA meta-regression accounting for ancestry with three axes of genetic variation as covariates; and (ii) MANTRA

Locus	Index SNP	MR-MEGA meta-regression			MANTRA		
		SNPs	Distance (bp)	Interval (bp)	SNPs	Distance (bp)	Interval (bp)
*IGF2BP2*	rs11705729	40	39,163	185,495,320–185,534,483	36	31,027	185,503,456–185,534,482
*CDKAL1*	rs9368222	6	12,330	20,675,792–20,688,121	5	12,330	20,675,792–20,688,121
*CDKN2A-B*	rs10965246	6	1,556	22,132,698–22,134,253	5	1,371	22,132,698–22,134,068
	rs10757282	6	4,041	22,133,645–22,137,685	7	4,435	22,133,251–22,137,685
*KCNQ1*	rs231353	4	38,477	2,691,471–2,729,947	3	17,549	2,691,471–2,709,019
	rs233448	13	20,175	2,837,723–2,857,897	11	20,273	2,837,625–2,857,897
	rs2237897	4	342	2,858,295–2,858,636	3	197	2,858,440–2,858,636

## Discussion

We have developed a novel approach to aggregating association summary statistics across GWAS from diverse populations through trans-ethnic meta-regression. The approach models allelic effects, weighted by their standard errors, as a function of axes of genetic variation, derived from pairwise allele frequency differences, genome-wide, between studies. Across a range of scenarios of heterogeneity in allelic effects between ancestry groups, meta-regression has increased power to detect association over fixed- and random-effects meta-analysis, whilst maintaining false positive error rates.

Axes of genetic variation are generated via multi-dimensional scaling of the mean allele frequency difference, genome-wide, between each pair of GWAS contributing to the meta-regression. In most consortia meta-analysis settings, allele frequencies would be expected to be provided as one of association summary statistics for each SNP, in addition to the allelic effect size and corresponding standard error, for example. If contributing GWAS do not provide allele frequency information, one solution is to use data from reference populations, such as those from the 1000 Genomes Project (13,14). GWAS from the same broad ethnic group would be matched to the same reference population, and would therefore be located at the same position on axes of genetic variation. Consequently, MR-MEGA would be able to detect heterogeneity in allelic effects between ancestry groups, but would not be able to recognise more subtle differences, due to admixture for example, within ethnicities. We would therefore expect there to be a relative loss in power to detect association in settings where heterogeneity in allelic effects was correlated with admixture proportions, for example in the ‘Native American’ scenario in our simulation study. However, we would still expect increased power over fixed- and random-effects analysis by allowing for heterogeneity between ethnic groups.

Alternative metrics to the genome-wide mean allele frequency difference exist for quantifying the extent of genetic differences between GWAS. We investigated the impact of an alternative metric, the fixation index (F_ST_) (18), on multi-dimensional scaling of the 26 populations from the 1000 Genomes Project Phase 3 reference panel (13) used in our simulation study. Whilst the absolute projection of populations onto the first three principal components changed from those obtained from mean allele frequency differences, their relative positions on these axes of genetic variation were highly correlated (Supplementary Material, Fig. S9). Consequently, the use of F_ST_ as a distance metric, instead of mean allele frequency differences, has no impact on our downstream meta-regression analysis results.

The meta-regression model assumes a linear trend in allelic effects with each axis of genetic variation included as a covariate. Whilst it is unlikely that this linear trend will hold exactly, we have demonstrated that axes of genetic variation are sufficient to cluster GWAS of similar ancestry, but also distinguish populations within the same ethnic group (Supplementary Materials, Figs S1, S3, S6). Consequently, if the allelic effect of a variant is specific to one ancestry, or varies between diverse populations according to their genetic similarity (within or between ethnic groups), including axes of genetic variation as covariates in the meta-regression model can account for this heterogeneity. Indeed, the heterogeneity scenarios considered in our simulation study do not assume a linear trend on the allelic effect of the causal variant in any of the axes of genetic variation (Supplementary Material, Table S1). However, in those scenarios for which heterogeneity is correlated (non-linearly) with ancestry (African specific, Eurasian and Native American), meta-regression including three axes of genetic variation as covariates offered improved power to detect association over fixed- and random-effects meta-analysis (Fig. 1). Only when heterogeneity is completely uncorrelated with ethnicity (non-ancestral scenario) did the power of the random-effects meta-analysis exceed that of the meta-regression.

The meta-regression model enables partitioning of heterogeneity in allelic effects between GWAS that is correlated with ancestry from residual variation due to other sources (such as variable phenotype definition). Heterogeneity in allelic effects due to ancestry is of particular relevance to fine-mapping, since it can occur as a result of differences in patterns of LD between diverse populations, which we model in the meta-regression framework by including axes of genetic variation as covariates. Consequently, the meta-regression model offers substantial gains in fine-mapping resolution over fixed- and random-effects meta-analysis, even for heterogeneity scenarios in which allelic effects do not follow a linear trend in the axes of genetic variation (Figs 2 and 3). We also compared the meta-regression approach with MANTRA, which models heterogeneity in allelic effects between GWAS according to a prior model of genetic similarity between them. The fine-mapping resolution achieved by the meta-regression model was greater than that for MANTRA, except in the scenario in which heterogeneity in allelic effects between studies was random, irrespective of ancestry, and cannot by accounted for by axes of genetic variation that distinguish broad ethnic groups. Similar performance between the methods was also observed through application to fine-mapping of association signals for T2D in four established susceptibility loci.

There has been recent development of novel methods for fine-mapping that utilise meta-analysis summary statistics and a reference panel of LD between variants across a locus, including CAVIAR (19), PAINTOR (10) and FINEMAP (20). By modelling LD between variants across a locus, these approaches have the advantage that they can allow for fine-mapping of multiple causal variants, simultaneously. However, CAVIAR and FINEMAP allow for specification of a single LD reference across the locus, which is not appropriate in the context of trans-ethnic fine-mapping because the correlation between variants is not the same for diverse populations. PAINTOR, on the other hand, overcomes this problem by allowing for specification of ethnic- or population-specific association summary statistics and LD references. Previously reported simulation highlighted substantial improvements in fine-mapping resolution for PAINTOR over an application of CAVIAR using an ‘average’ LD reference across all ethnic groups (10). PAINTOR also has the advantage that it can incorporate a prior model of causality based on genomic annotation, allowing a boost in the posterior probability that coding variants drive association signals, for example, as observed in genome-wide enrichment analyses (21). Nevertheless, the results of our simulation study of loci with a single causal variant highlight that PAINTOR is not well calibrated across the scenarios considered, even in the ‘perfect’ data setting, and has lower resolution than MR-MEGA and MANTRA (larger 99% credible sets and less posterior probability ascribed to the causal variant) when heterogeneity in allelic effects is correlated with ancestry.

An alternative approach to allow for multiple causal variants is to first dissect ‘distinct’ association signals at a GWAS locus through (approximate) conditional analysis (22). Conditional analyses can be performed using backward elimination to identify index variants for each distinct association signal, for example as implemented in GCTA (23), until association at the locus is fully explained. Fine-mapping is then undertaken for each distinct association signal by conditioning on all other index variants at the locus. Each of these distinct signals is assumed to represent a different underlying causal variant, acting in isolation or through haplotype effects. Such an approach has been widely employed for fine-mapping association signals for a range of complex human traits and diseases, in the context of both trans-ethnic and ancestry-specific meta-analyses (11,12,17,24–32).

Unfortunately, the results of our simulation study highlight that there is no single optimal approach to the aggregation of GWAS from diverse populations across the range of scenarios for heterogeneity in allelic effects we have considered. For example, under a scenario in which allelic effects are homogeneous across ethnic groups, there is a small loss in power for the meta-regression model compared to fixed- and random-effects meta-analysis that is due to the inclusion of axes of genetic variation as covariates that are not predictive of heterogeneity. Our analyses have focussed on three axes that distinguish populations of African, East Asian, European, Native American and South Asian ancestry. Reducing the number of axes of genetic variation included as covariates in the meta-regression model would decrease the loss in power, compared to fixed- or random-effects meta-analysis, under a scenario of homogenous allelic effects across populations. However, the power of the meta-regression model to detect SNP association would then be decreased when heterogeneity in allelic effects between GWAS is driven by ancestry. One solution to this dilemma is to use both fixed-effects meta-analysis and meta-regression for aggregation of GWAS from diverse populations, although thresholds of significance should be adjusted to account for multiple testing at each SNP.

One of the advantages of the meta-regression approach is that we can assess the contribution of each axis of genetic variation to heterogeneity in allelic effects between GWAS. For example, we observed strong evidence for heterogeneity in allelic effects on T2D susceptibility due to ancestry at the *CDKAL1* locus, which was accounted for by one axis of genetic variation. Allelic effect sizes increased along this axis, separating those of South Asian ancestry from other ethnic groups, consistent with previous reports that this locus has greater impact on populations of European and East Asian descent.

A second advantage of the meta-regression approach is that additional covariates can be included to investigate other sources of potential heterogeneity in allelic effects between studies. For example, where sex-specific association summary statistics are available, inclusion of sex as covariate provides an assessment in allelic effects between males and females, after accounting for ancestry. Inclusion of imputation quality metrics as a covariate enables confirmation that apparent heterogeneity in allelic effects between studies is not a reflection of variable imputation success, which may vary according to ancestry because of the availability of closely matched population haplotypes in the reference panel, for example.

In conclusion, trans-ethnic meta-regression, as implemented in the MR-MEGA software, offers a powerful approach for the discovery and fine-mapping of complex trait loci across GWAS from diverse populations. With the increasing availability of multi-ancestry GWAS of complex human traits, powerful statistical methodology for trans-ethnic meta-analysis, such as that implemented in MR-MEGA, shows great promise for future improvements in our understanding of the genetic basis of common diseases.

## Materials and Methods

Consider a series of *K* GWAS of a complex trait. At each variant, we assume that all GWAS are aligned to the same reference allele. We denote the reference allele frequency of the *j*th SNP in the *k*th GWAS by *p_kj_*. We construct a matrix of pairwise Euclidean distances between GWAS across autosomal variants, denoted ***D*** = [*d_kk_*_’_], where

$$d_{kk'}=\frac{\sum_{j}I_{j}{\left(p_{kj}-p_{k'j}\right)}^{2}}{\sum_{j}I_{j}}.$$

In this expression, *I_j_* is a binary indicator variable of the inclusion of the *j*th variant in the distance calculation. We recommend dividing the genome into 1 Mb bins, and utilising one variant with MAF of at least 5% in all GWAS from each bin to minimise the impact of LD. We then implement multi-dimensional scaling of the distance matrix, ***D***, to derive *T* axes of genetic variation, denoted ***x***_*k*_ for the *k*th GWAS. Note that the choice of the number of axes of genetic variation will depend on the population diversity of GWAS, but is restricted to *T *≤* K*-2.

For the *j*th variant, we denote the estimated effect of the reference allele in the *k*th GWAS, and the corresponding variance, by *b_kj_* and *v_kj_*, respectively. We then model the reference allele effect across GWAS in a linear regression model, given by

(1) $$E\left(b_{kj}\right)=\alpha_{j}+\sum_{t=1}^{T}\beta_{tj}x_{kt},$$

where *α_j_* is the intercept and *β_tj_* is the effect of the *t*th axis of genetic variation for the *j*th variant. The contribution of the *k*th GWAS is weighted by the estimated inverse variance of the reference allele effect at the *j*th variant, denoted $v_{kj}^{-1}$. We can interpret the intercept, *α_j_*, as the expected allelic effect of the *j*th variant for a population of ancestry represented by zero on each of the *T* axes of genetic variation.

We test the null hypothesis of no association of the *j*th variant across GWAS by comparing the deviance of model (1) with *α_j_* = *β*_1__*j*_ = … = *β_Tj_* = 0 to that for which the parameters are unconstrained, with the resulting test statistic denoted *X_j_* having an approximate chi-squared distribution with *T *+* *1 degrees of freedom. We can also test for the presence of heterogeneity in allelic effects between GWAS that is correlated with ancestry by comparing the deviance of model (1) with *β*_1__*j*_ = … = *β_Tj_* = 0 to that for which the parameters are unconstrained, with the resulting test statistic having an approximate chi-squared distribution with *T* degrees of freedom. Finally, the deviance of model (1), with all parameters unconstrained, provides a test of residual heterogeneity in allelic effects between GWAS after accounting for ancestry, having an approximate chi-squared distribution with *K*-*T*-1 degrees of freedom.

We can also assess the contribution of the *t*th axis of genetic variation to heterogeneity in allelic effects by comparing the deviance of model (1) with *β_tj_* = 0 to that for which the parameters are unconstrained, with the resulting test statistic having an approximate chi-squared distribution with one degree of freedom.

### Fine-mapping

Consider a locus encompassing a pre-specified interval from an index variant. For the *j*th variant in the locus, we approximate the Bayes’ factor in favour of association (33) by

$$\Lambda_{j}=exp\left[\frac{X_{j}-\left(T+1\right)lnK}{2}\right].$$

We then calculate the posterior probability that the *j*th variant is driving the association signal at the locus by

$$\pi_{j}=\frac{\Lambda_{j}}{\sum_{i}\Lambda_{i}}.$$

In this expression, the summation in the denominator is over all variants across the locus. Finally, we derive a 99% credible set (34) for the association signal by: (i) ranking all variants according to their Bayes’ factor, *Λ_j_*; and (ii) including ranked variants until their cumulative posterior probability of driving the association attains or exceeds 0.99.

### Software

We have implemented the methodology in the MR-MEGA (Meta-Regression of Multi-Ethnic Genetic Association) software (http://www.geenivaramu.ee/en/tools/mr-mega). For each study, a flat file of association summary statistics is required, including one row per variant, and columns for the variant name and position in the genome, effect and other alleles, effect allele frequency, allelic effect and standard error, and sample size. For each variant, MR-MEGA aligns studies to the same effect allele, and flips the allele frequency and allelic effect if required. MR-MEGA then performs multi-dimensional scaling of mean genome-wide allele frequency differences between each pair of GWAS. Meta-regression is undertaken in a linear regression framework, as described above, including axes of genetic variation as covariates in the model. MR-MEGA can perform genomic control at the study level, and/or after meta-regression. For each variant, MR-MEGA provides: (i) *P*-value and Bayes’ factor in favour of association, accounting for heterogeneity that is correlated with ancestry; (ii) *P*-value for heterogeneity that is correlated with ancestry; and (iii) *P*-value for residual heterogeneity.

### Simulation study: false positive error rate and power

For each replicate, the causal variant was selected at random from those reported in the reference panel from Phase 3 of the 1000 Genomes Project (13) with MAF > 1% in all populations. Genotypes in each population were then simulated using the causal variant population-specific odds-ratio (Supplementary Material, Table S1) and 1000 Genomes Project allele frequency, under an assumption of Hardy-Weinberg equilibrium. For each replicate of data, in each population, we tested for association of the causal variant with case-control status in a logistic regression framework under an additive model in the log-odds ratio in PLINK (35), and obtained estimated allelic effect sizes, corresponding standard errors and Z-scores.

We then tested for association of the causal variant with case-control status across populations using the meta-regression model, implemented in MR-MEGA, including three axes of genetic variation as covariates to separate ancestry groups. For comparison, we also tested for association using fixed-effects (inverse-variance weighted log-odds ratios) and random-effects (RE2) meta-analysis implemented in METASOFT (7). False positive error rates were assessed at a nominal significance threshold (*P *<* *0.05), whilst power was evaluated at genome-wide significance (*P *<* *5×10^−8^). Next, we tested for heterogeneity, at nominal significance (*P *<* *0.05), in allelic effects between populations that is correlated with ancestry using the meta-regression model implemented in MR-MEGA. Finally, we tested for residual heterogeneity, at nominal significance (*P *<* *0.05), in allelic effects between populations: (i) from the meta-regression model implemented in MR-MEGA after accounting for ancestry; and (ii) using Cochran’s *Q* statistic from the fixed-effects meta-analysis implemented in METASOFT (7).

### Simulation study: fine-mapping loci with a single causal variant

For each replicate, the 2 Mb region was centred on a single causal variant, selected at random from those reported in the reference panel from Phase 3 of the 1000 Genomes Project (13) with MAF > 1% in all reference populations. Genotypes in each population were then simulated, using HAPGEN2 (36), using the causal variant population-specific odds-ratio (Supplementary Material, Table S1) and haplotypes from the 1000 Genomes Project Phase 3 reference panel (13).

We first considered the ‘perfect’ data setting, where all variants in the region are captured, with no missing genotypes or errors, for benchmarking purposes. For each replicate of data, we considered the 1 Mb region centred on the causal variant. Within each population, we tested all variants in this region for association with case-control status in a logistic regression framework under an additive model in the log-odds ratio in PLINK (35), and obtained estimated allelic effect sizes, corresponding standard errors and Z-scores.

We then considered the more realistic ‘imperfect’ data setting. For each replicate of data, genotypes at only 100 randomly selected variants in the 2 Mb region were retained, to represent a typical GWAS array. Within each population, separately, this scaffold of genotypes was imputed up to haplotypes from the 1000 Genomes Project Phase 3 reference panel (13) using IMPUTEv2 (37,38). Imputation was performed in the 1 Mb region centred on the causal variant, with the remaining 500 kb regions up- and down-stream retained as buffers. Within each population, we then tested for association of all variants with case-control status in a logistic regression framework under an additive model in the log-odds ratio in SNPTEST (39), taking account of uncertainty in the imputation process with the genotype dosage (‘expected’ option), and obtained estimated allelic effect sizes, corresponding standard errors and Z-scores. We performed post-imputation quality control, and excluded variants with IMPUTEv2 info < 0.4 from downstream analyses (40).

For each replicate of data, for both ‘perfect’ and ‘imperfect’ data settings, we obtained Bayes’ factors in favour of association for each variant from the meta-regression model, implemented in MR-MEGA, including three axes of genetic variation as covariates to separate ancestry groups. For comparison, we also obtained, for each variant: (i) approximate Bayes’ factors (41) on the basis of allelic effect estimates, and corresponding standard errors, from fixed-effects and random-effects meta-analysis implemented in METASOFT (7), assuming a Gaussian prior N(0,0.2^2^) for log-odds ratios; and (ii) the Bayes’ factor from MANTRA (8) using the matrix of pairwise Euclidean distances between the reference populations to model heterogeneity. These (approximate) Bayes’ factors were used to obtain the posterior probability of driving the association for each variant across the locus. Finally, we undertook trans-ethnic meta-analysis across populations using PAINTOR (10), assuming a single causal variant at the locus (option ‘-enumerate 1’), and approximating LD between variants in each population from haplotypes in the 1000 Genomes Project Phase 3 reference panel (13). Under the assumption of a uniform prior model of causality (no functional enrichment), we used PAINTOR to generate the posterior probability of driving the association for each variant across the locus. In each replicate, we constructed the 99% credible set driving the association signal at the locus for each method by: (i) ranking all variants by their posterior probability; and (ii) including ranked variants until their cumulative posterior probability attains or exceeds 0.99.

### Trans-ethnic meta-analysis of GWAS of kidney function

Each GWAS was pre-phased and imputed up to the 1000 Genomes Project Phase 1 reference panel (14) using IMPUTEv2 (37,38) or minimac (37). Variants were retained for analysis in each GWAS if: (i) MAF ≥ 0.5%; and (ii) IMPUTEv2 info ≥ 0.4 or minimac *r*^2^≥0.3 (40). Kidney function was assessed by eGFR, calculated from serum creatinine (mg/dL), with adjustment for age, sex and ethnicity by means of the four variable Modification of Diet in Renal Disease equation (42). Within each study, association of eGFR with each variant was tested in a linear regression framework, under an additive dosage model, and with adjustment for study-specific covariates to account for confounding due to population structure (Supplementary Material, Table S5). Within each study, association summary statistics were corrected in each study for residual population structure by genomic control (43) (Supplementary Material, Table S5).

Association summary statistics for each variant passing quality control in at least 50% of the total sample size were aggregated across studies via fixed-effects meta-analysis, with inverse-variance weighting, implemented in METASOFT (7). Association summary statistics from the meta-analysis were then corrected for a second round of genomic control (43) (*λ*_GC_=1.029). Heterogeneity in allelic effects between studies at each variant was assessed by means of Cochran’s *Q*-statistic from the fixed-effects meta-analysis implemented in METASOFT (7). We implemented multi-dimensional scaling of the matrix of pairwise Euclidean distances between studies to derive two axes of genetic variation that were sufficient to separate GWAS between ancestry groups (Supplementary Material, Fig. S3). We then applied the meta-regression model, implemented in MR-MEGA, to each variant passing quality control in at least 50% of the total sample size, including the two axes of genetic variation as covariates. Association summary statistics from the meta-analysis were then corrected for a second round of genomic control (43) (*λ*_GC_=1.017).

### Fine-mapping of four T2D susceptibility loci: *CDKAL1*, *CDKN2A-B*, *IGF2BP2* and *KCNQ1*

We made use of summary statistics derived by the T2D-GENES Consortium (12) for seven distinct signals of T2D association at the four loci. Briefly, at each locus, the scaffold of genome-wide genotype data in each GWAS was imputed up to the 1000 Genomes Project Phase 1 reference panel (14) using IMPUTEv2 (37,38) or minimac (37). Variants were retained for analysis in each study if: (i) MAF ≥ 1%; and (ii) IMPUTEv2 info ≥ 0.4 or minimac *r*^2^ ≥ 0.3 (40). These variants were used to derive a matrix of pairwise Euclidean distances between the studies. T2D association with each retained variant was tested in a logistic regression framework under an additive model in the log-odds ratio, and estimated allelic effect sizes and corresponding standard errors were obtained. Association summary statistics for each variant passing quality control in at least 80% of the total sample size were then aggregated across GWAS using MANTRA (8) using the matrix of pairwise Euclidean distances between studies. Step-wise conditional analyses were undertaken at each locus, at each stage including the variant with the strongest association as a covariate until the residual signal did not attain genome-wide significance (MANTRA log_10_ Bayes’ factor >6).

We implemented multi-dimensional scaling of the Euclidean distance matrix to derive three axes of genetic variation to separate GWAS between ancestry groups (Supplementary Material, Fig. S6). For each distinct association signal, we applied the meta-regression model, implemented in MR-MEGA, including the three axes of genetic variation as covariates, to each variant passing quality control in at least 80% of the total sample size. From this model, we assessed the evidence of T2D association for each SNP and the extent of heterogeneity in allelic effects between GWAS that is correlated with ancestry. Subsequently, we obtained a Bayes’ factor in favour of T2D association and constructed a 99% credible set of variants driving each of the distinct signals.

## Supplementary Material

Supplementary Material is available at *HMG* online.

*Conflict of Interest statement.* None declared. 

## Supplementary Material

Supplementary Figures and Tables
