# Supplementary material for: Trans-ethnic meta-regression of genome-wide association studies accounting for ancestry increases power for discovery and improves fine-mapping resolution
Source: Hum Mol Genet. 2017 Jul 17;26(18):3639–50. doi: 10.1093/hmg/ddx280 (PMC5755684; doi:10.1093/hmg/ddx280)
Supplement: Supplementary Figures and Tables [file supplementary_material_ddx280.pdf]

Figure S1. Axes of genetic variation separating 26 reference populations from Phase 3 of the 1000 Genomes Project. The first three axes of genetic variation from multi-dimensional scaling of the Euclidean distance matrix between populations are sufficient to separate five ancestry groups: African (AFR), Native American (AMR), East Asian (EAS), European (EUR) and SAS (South Asian). Population codes are presented in **Table S1**.

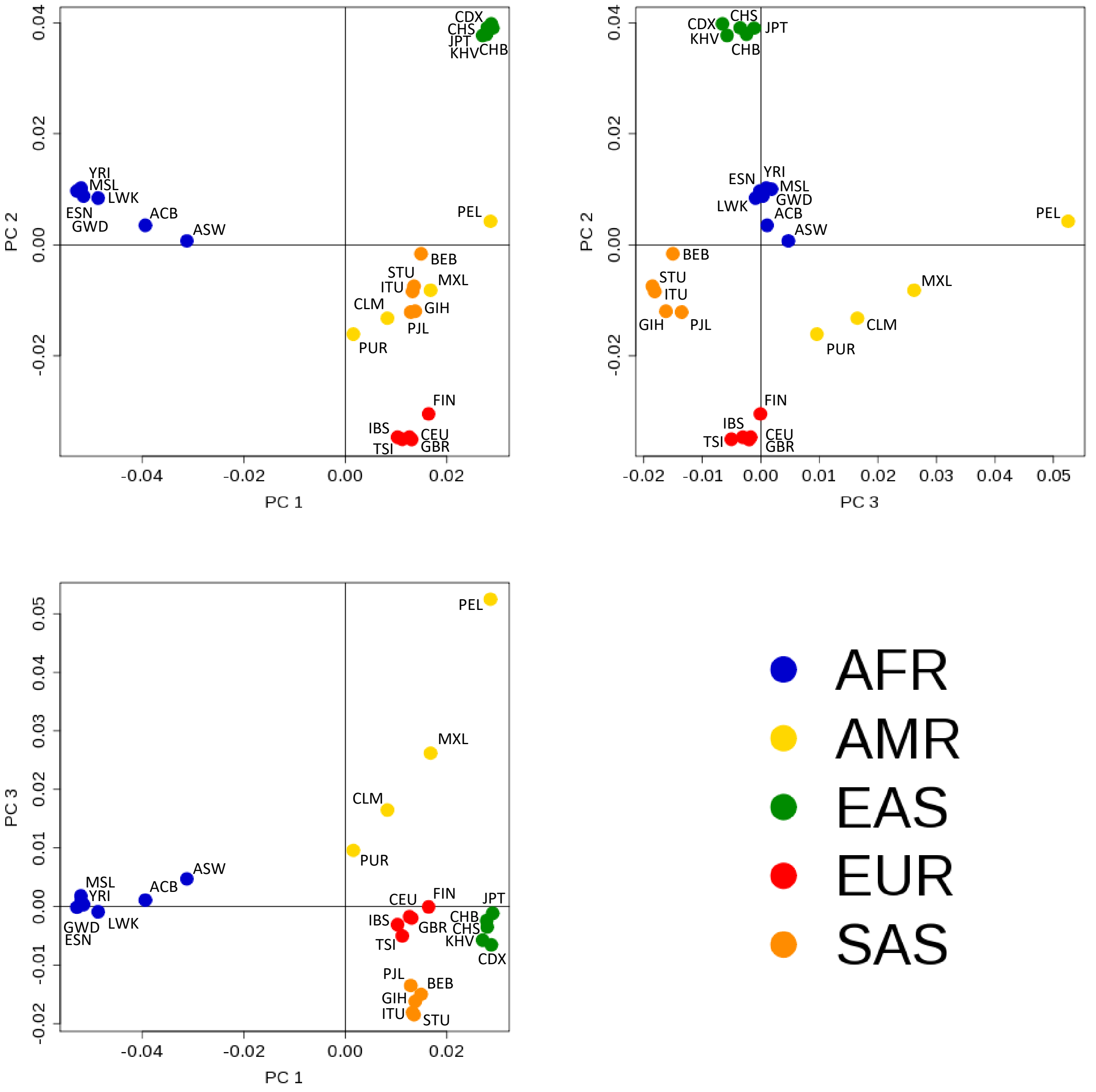

Figure S2. Power to detect, at nominal significance (*p*<0.05): heterogeneity in allelic effects between studies due to ancestry from MR-MEGA; residual heterogeneity in allelic effects between studies after accounting for ancestry from MR-MEGA; and heterogeneity in allelic effects between studies via Cochran's *Q* statistic from fixed-effects meta-analysis. Power is presented as a function of the allelic odds-ratio for each of five scenarios for heterogeneity in effects between populations, described in **Table S1**.

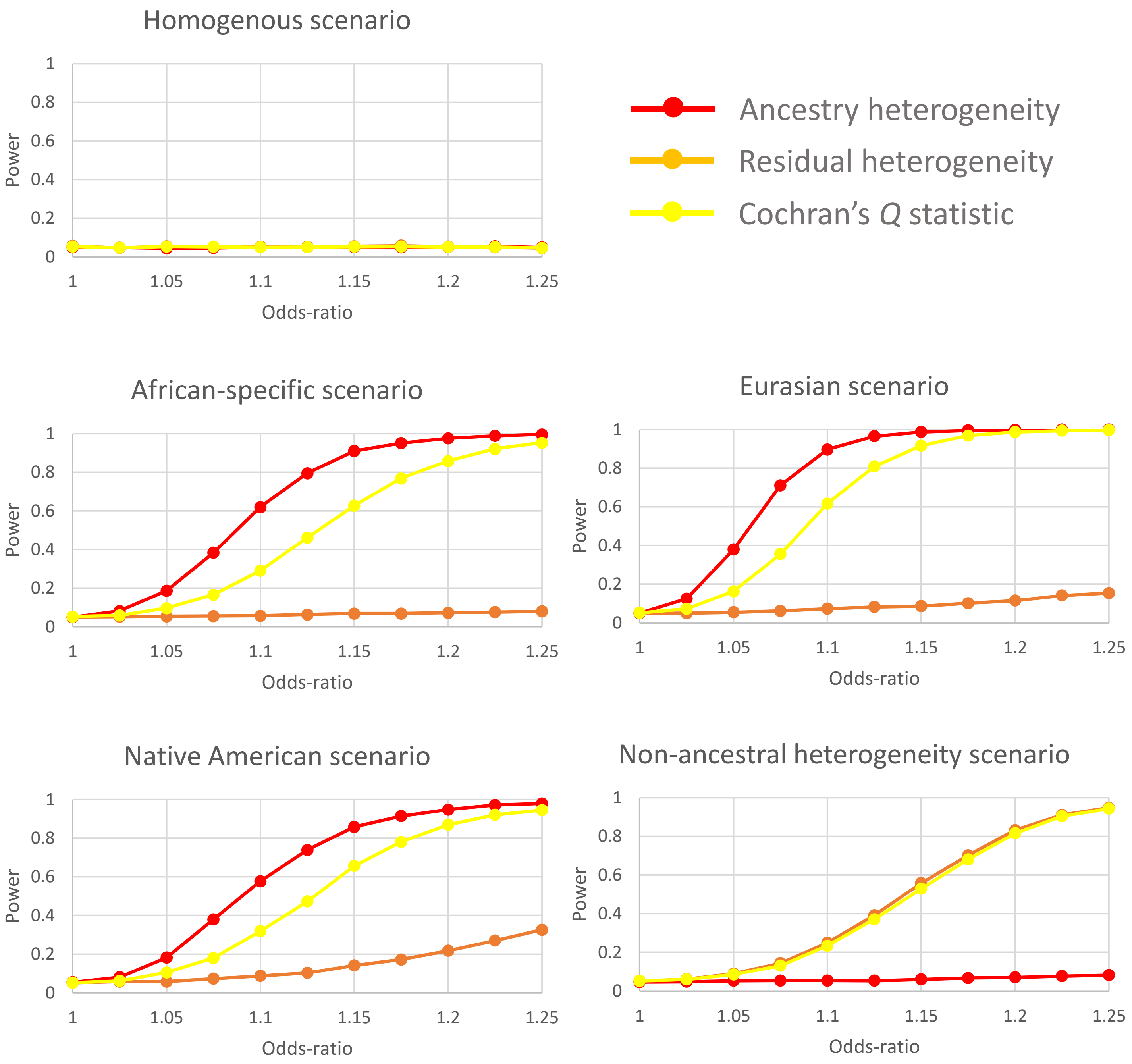

Figure S3. Axes of genetic variation separating nine GWAS of eGFR from the COGENT-Kidney Consortium. The first two axes of genetic variation from multi-dimensional scaling of the Euclidean distance matrix between GWAS are sufficient to separate four ancestry groups: African American (AFA), Hispanic/Latino (HIS), East Asian (EAS) and European (EUR).

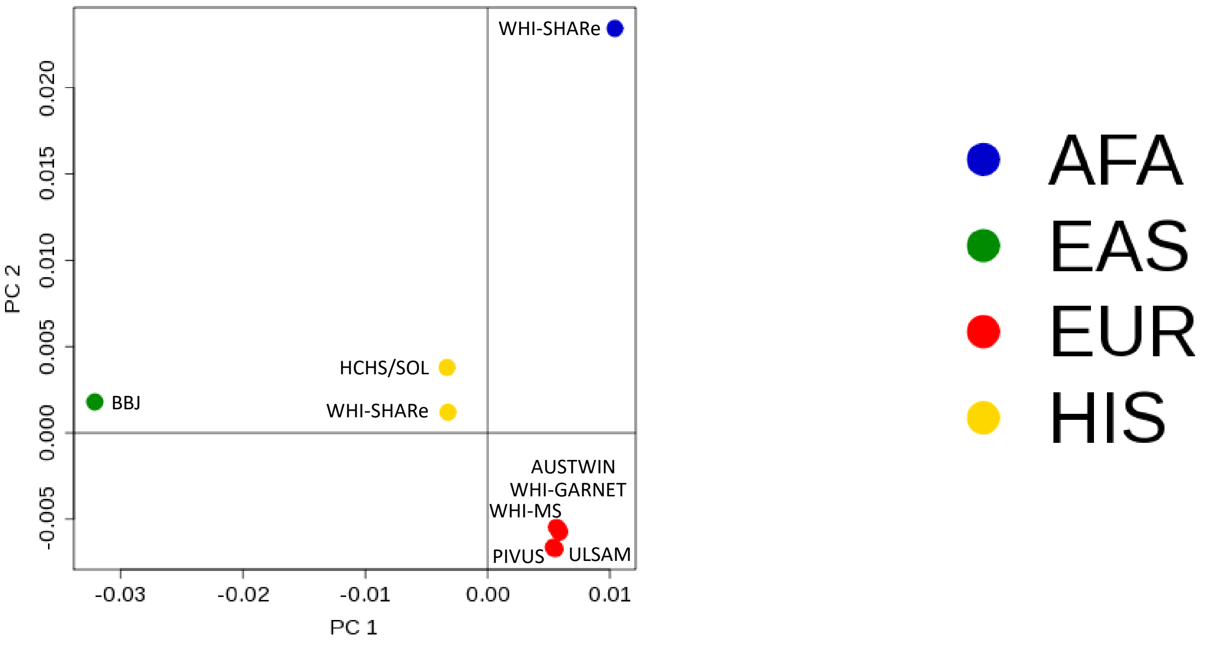

Figure S4. Comparison of p-values obtained for association with eGFR in trans-ethnic meta-analysis of 71,461 individuals from the COGENT-Kidney Consortium from meta-regression (MR-MEGA) and fixed-effects (inverse-variance weighting). Points are coloured according to the p-value for heterogeneity correlated with ancestry from the meta-regression: $p<0.05$ (red) and $p\geq 0.05$ (blue).

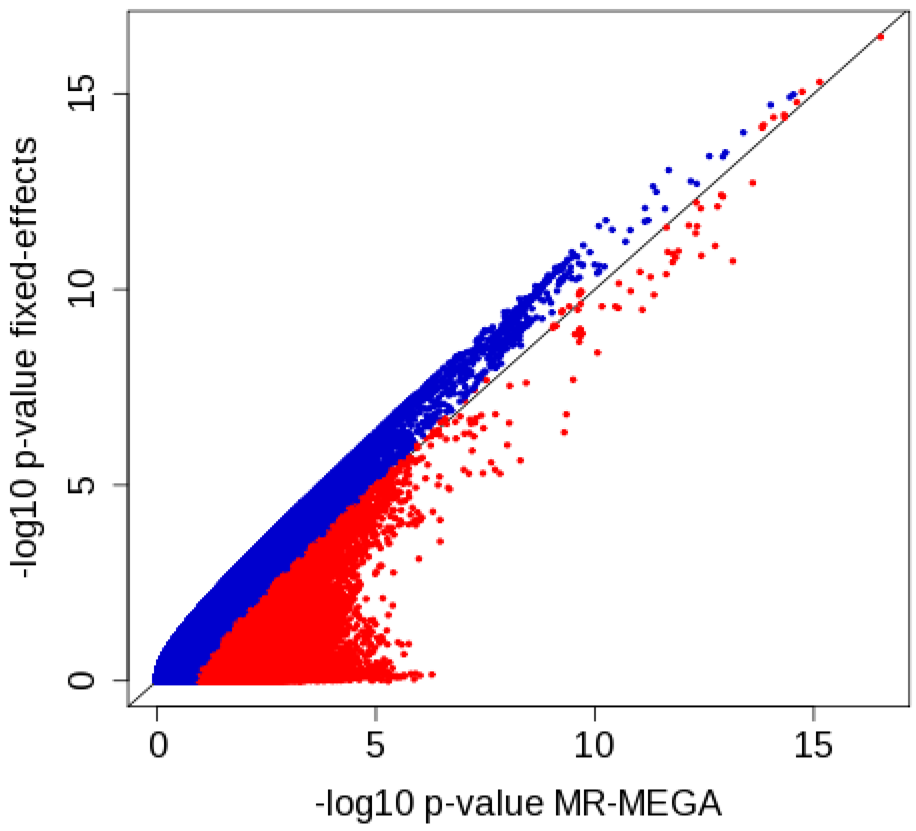

Figure S5. Forest plot of allelic effects for eGFR at the lead SNP (rs690428) at the *WDR72* locus across GWAS in trans-ethnic meta-analysis of 71,461 individuals from the COGENT-Kidney Consortium. Allelic effects are aligned to allele A across GWAS: European ancestry (PIVUS, ULSAM, AUSTWIN, WHI-GARNET and WHI-MS), East Asian ancestry (BBJ), Hispanic/Latino ancestry (HCHS/SOL and WHI-SHARe-HIS) and African American ancestry (WHI-SHARe-AFA).

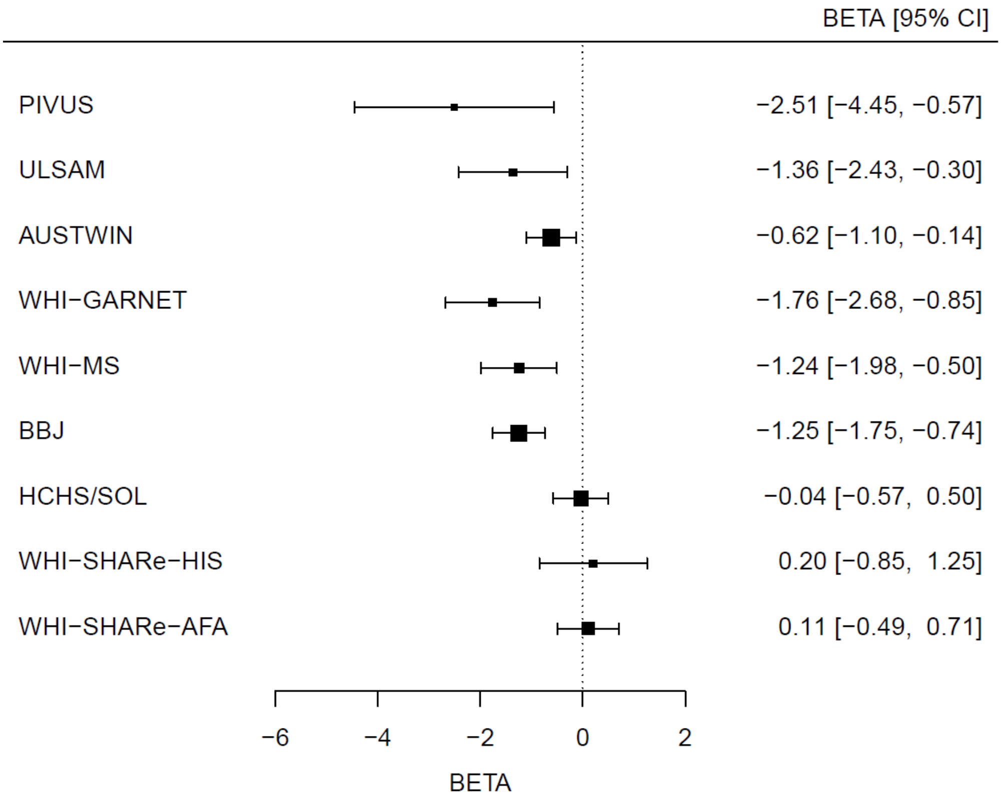

Figure S6. Axes of genetic variation separating 18 GWAS of T2D susceptibility from the T2D-GENES Consortium. The first three axes of genetic variation from multi-dimensional scaling of the Euclidean distance matrix between GWAS are sufficient to separate five ancestry groups: African American (AFR), Mexican American (AMR), East Asian (EAS), European (EUR) and SAS (South Asian).

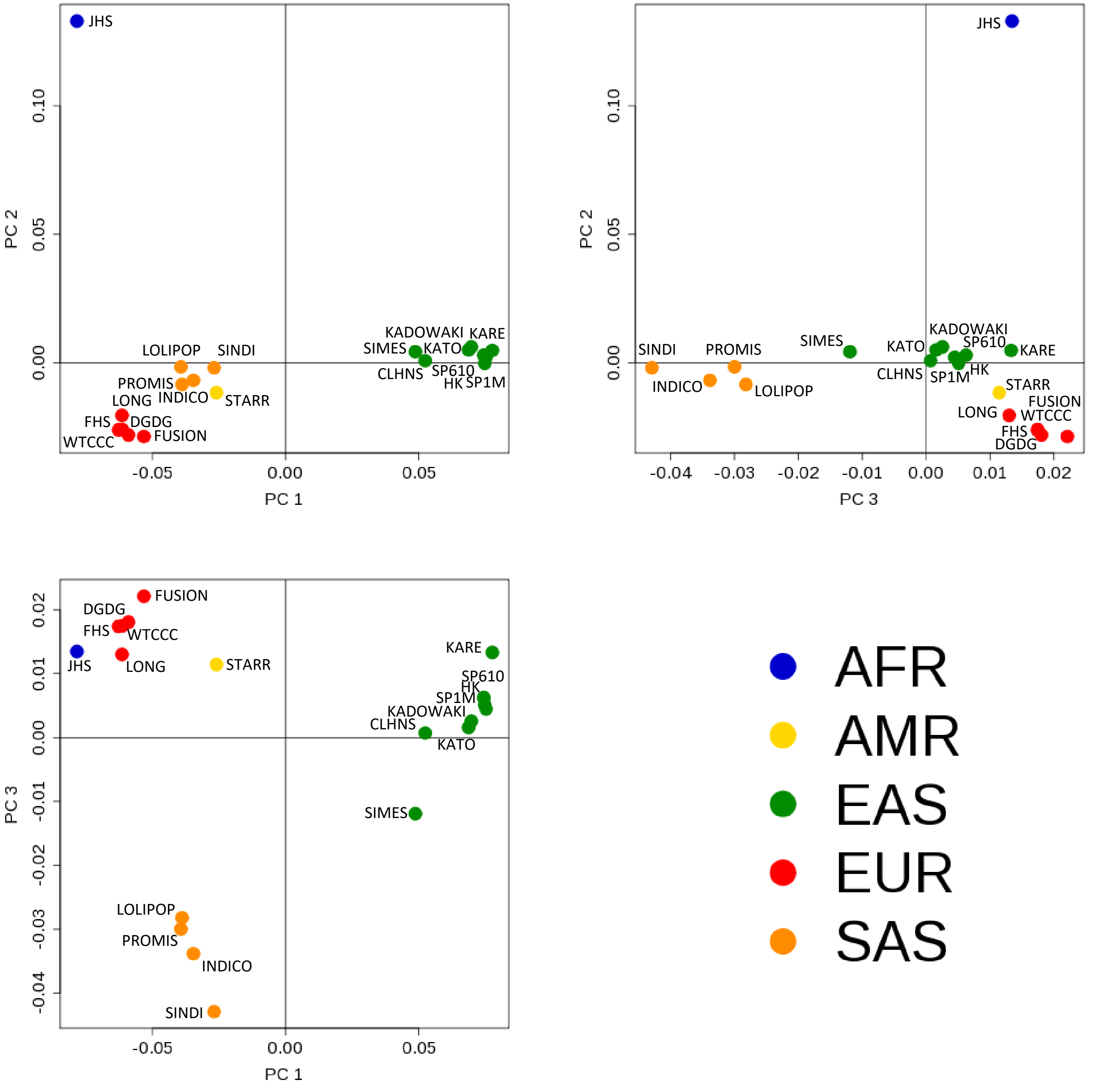

Figure S7. Signal plots for seven distinct association signals at four T2D susceptibility, constructed on the basis of aggregation of summary statistics from 18 GWAS (22,086 cases and 42,539 controls) from diverse populations using meta-regression accounting for ancestry with three axes of genetic variation as covariates. Each point represents a SNP passing quality control in the meta-regression, plotted with their $\log_{10}$ *p*-value as a function of genomic position (NCBI build 37). In each plot, the index SNP is represented by the purple symbol. The colour coding of all other SNPs indicates LD with the index SNP (estimated by EUR r^2 from 1000 Genomes Project reference haplotypes): red $r^2 \geq 0.8$; gold $0.6 \leq r^2 < 0.8$; green $0.4 \leq r^2 < 0.6$; cyan $0.2 \leq r^2 < 0.4$; blue $r^2 < 0.2$; grey r^2 unknown. Recombination rates are estimated from Phase II HapMap and gene annotations are taken from the UCSC genome browser.

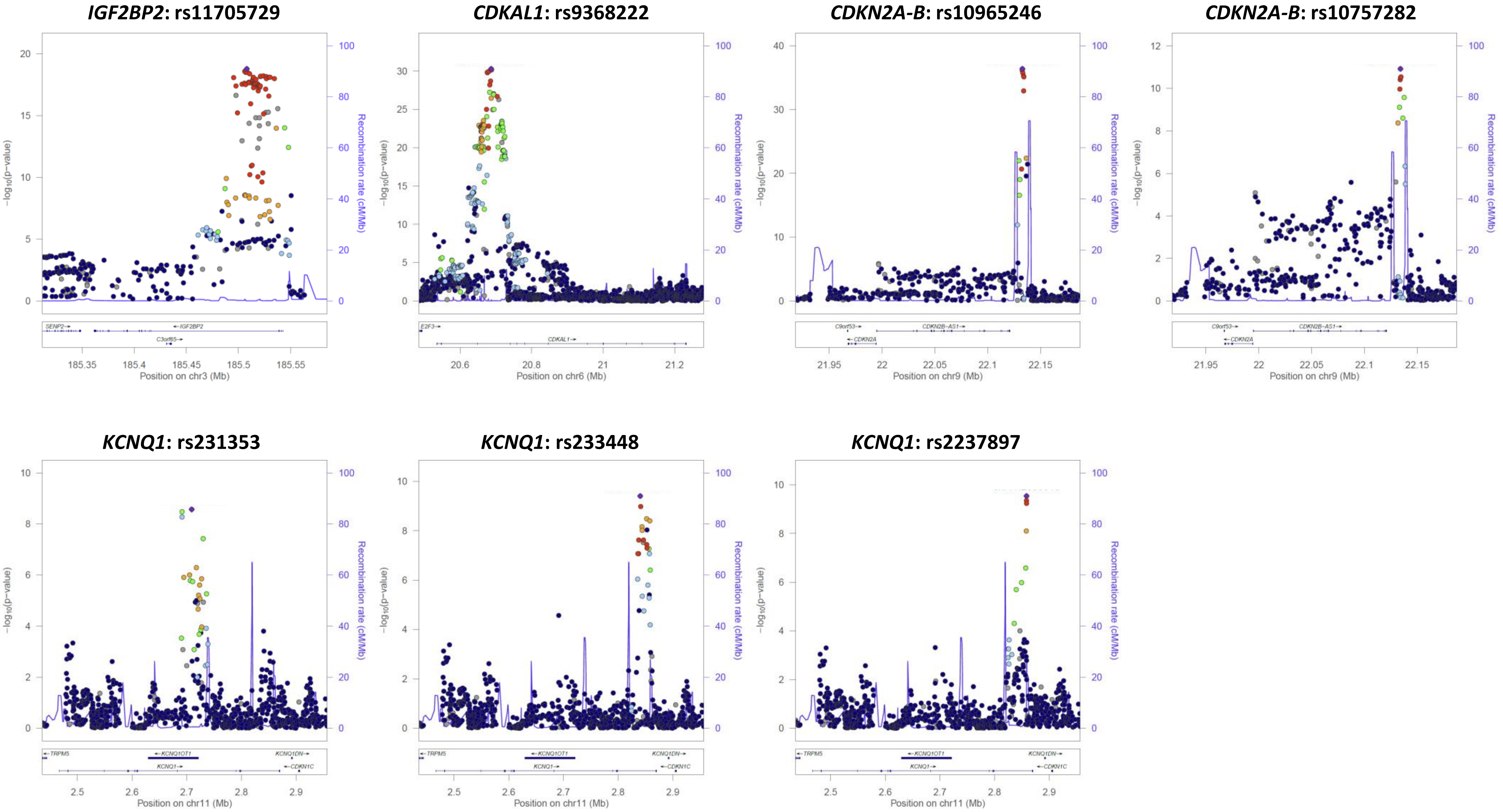

Figure S8. T2D allelic log-odds ratios for the index SNP, rs9368222, at the *CDKAL1* locus across 18 GWAS (22,086 cases and 42,539 controls) from diverse populations. Effect sizes in each GWAS are plotted according to their position on the third axis of genetic variation (PC 3). Grey bars represent 95% confidence intervals for log-ORs. Each GWAS is coloured according to ancestry: African American (blue); East Asian (green); European (red); Hispanic (yellow); and South Asian (orange).

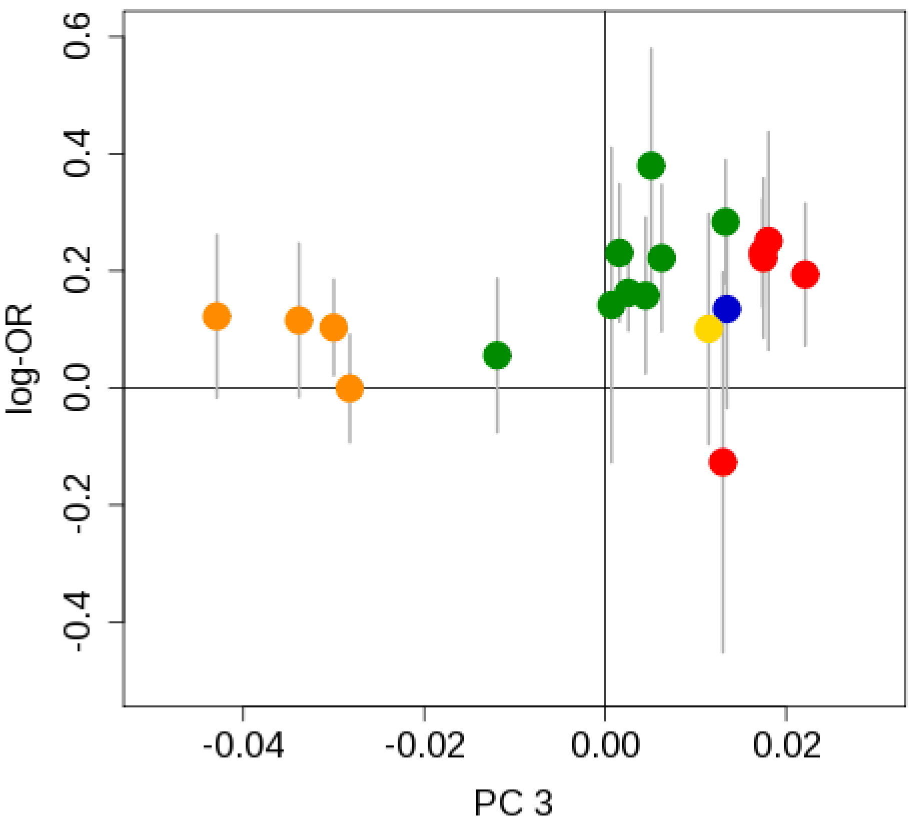

Figure S9. Comparison of axes of genetic variation obtained from multi-dimensional scaling of pair-wise distances between populations from the Phase 3 1000 Genomes Project reference panel: (i) mean genome-wide allele frequency difference, implemented in MR-MEGA; and (ii) fixation index (F_{ST}). Each point represents a population, plotted according to their position on the first three axes of genetic variation (PC1, PC2 and PC3) obtained from the two metrics.

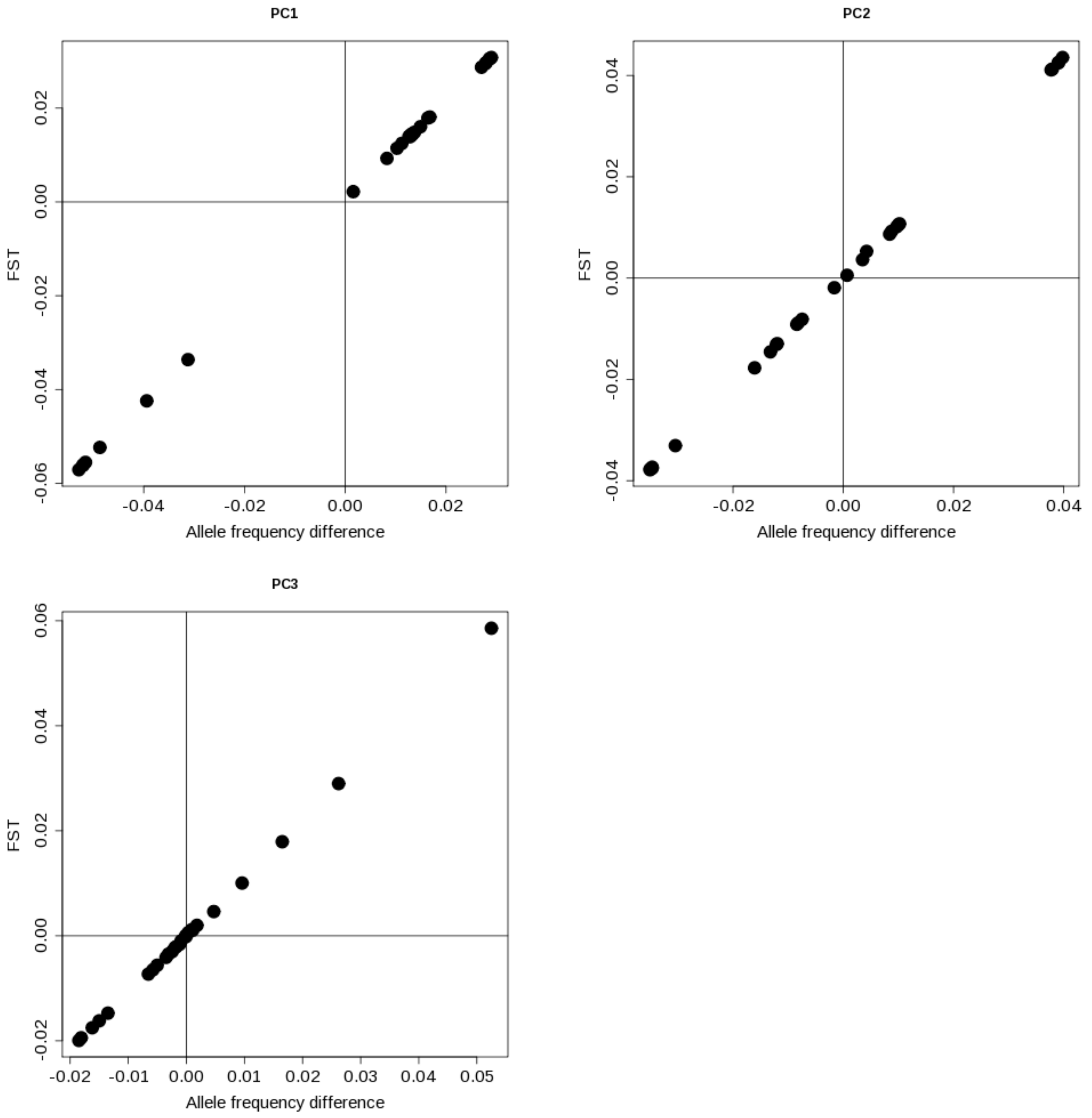

Table S1. Heterogeneity scenarios parameterised in terms of the allelic odds-ratio, ψ, in each reference population from Phase 3 of the 1000 Genomes Project.

Population			Heterogeneity scenario				
**Code**	**Description**	**Ancestry**	**Homogeneous**	**African-specific**	**Eurasian**	**Native American**	**Non-ancestral**
ACB	African Caribbean in Barbados	African	ψ	ψ	1	1	ψ
ASW	African Ancestry in Southwest USA	African	ψ	ψ	1	1	1
ESN	Esan in Nigeria	African	ψ	ψ	1	1	1
GWD	Gambian in Western Division, The Gambia	African	ψ	ψ	1	1	1
LWK	Luhya in Webuye, Kenya	African	ψ	ψ	1	1	1
MSL	Mende in Sierra Leone	African	ψ	ψ	1	1	1
YRI	Yoruba in Ibadan, Nigeria	African	ψ	ψ	1	1	1
CLM	Colombian in Medellin, Colombia	Native American	ψ	1	ψ	ψ	ψ
MXL	Mexican Ancestry in Los Angeles, California	Native American	ψ	1	ψ	ψ	1
PEL	Peruvian in Lima, Peru	Native American	ψ	1	ψ	2ψ	1
PUR	Puerto Rican in Puerto Rico	Native American	ψ	1	ψ	ψ	1
CDX	Chinese Dai in Xishuangbanna, China	East Asian	ψ	1	2ψ	1	ψ
CHB	Han Chinese in Bejing, China	East Asian	ψ	1	2ψ	1	1
CHS	Southern Han Chinese in China	East Asian	ψ	1	2ψ	1	1
JPT	Japanese in Tokyo, Japan	East Asian	ψ	1	2ψ	1	1
KHV	Kinh in Ho Chi Minh City, Vietnam	East Asian	ψ	1	2ψ	1	1
CEU	Northern/Western European ancestry in Utah	European	ψ	1	ψ	1	ψ
FIN	Finnish in Finland	European	ψ	1	ψ	1	1
GBR	British in England and Scotland	European	ψ	1	ψ	1	1
IBS	Iberian populations in Spain	European	ψ	1	ψ	1	1
TSI	Toscani in Italy	European	ψ	1	ψ	1	1
BEB	Bengali in Bangladesh	South Asian	ψ	1	ψ	1	ψ
GIH	Gujarati Indian in Houston, Texas	South Asian	ψ	1	ψ	1	1
ITU	Indian Telugu in the UK	South Asian	ψ	1	ψ	1	1
PJL	Punjabi in Lahore, Pakistan	South Asian	ψ	1	ψ	1	1
STU	Sri Lankan Tamil in the UK	South Asian	ψ	1	ψ	1	1

Table S2. False positive error rates (standard error), at a nominal significance threshold (*p*<0.05), to detect SNP association across a range of heterogeneity scenarios on the basis of aggregation of association summary statistics from 26 GWAS (each of 1,000 cases and 1,000 controls) from diverse populations using: (i) fixed-effects (inverse-variance weighted log-odds ratios) meta-analysis; (ii) random-effects (RE2) meta-analysis; and (iii) meta-regression accounting for ancestry with three axes of genetic variation as covariates.

Heterogeneity scenario	Fixed-effects meta-analysis	Random-effects meta-analysis	Meta-regression
Homogeneous	0.0482 (0.0068)	0.0468 (0.0067)	0.0478 (0.0067)
African-specific	0.0540 (0.0071)	0.0506 (0.0069)	0.0512 (0.0070)
Eurasian	0.0492 (0.0068)	0.0470 (0.0067)	0.0460 (0.0066)
Native American	0.0480 (0.0068)	0.0472 (0.0067)	0.0504 (0.0069)
Non-ancestral	0.0492 (0.0068)	0.0496 (0.0069)	0.0480 (0.0068)

Table S3. Coverage of the causal variant by the 99% credible set across 500 simulations of each scenario with imputed data for five fine-mapping approaches: (i) fixed-effects meta-analysis; (ii) random-effects meta-analysis; (iii) meta-regression accounting for heterogeneity in allelic effects implemented in MR-MEGA; (iv) MANTRA; and (v) PAINTOR.

**Fine-mapping method**	**Heterogeneity scenario**				
	**Homogeneous**	**African-specific**	**Eurasian**	**Native American**	**Non-ancestral**
Fixed-effects	0.976	0.966	0.614	0.924	0.966
Random-effects	0.974	0.964	0.938	0.966	0.966
Meta-regression	0.962	0.96	0.846	0.892	0.92
MANTRA	0.966	0.936	0.836	0.808	0.884
PAINTOR	0.544	0.698	0.802	0.778	0.77

Table S4. Mean run times for five fine-mapping methods to assess association with variants within a 1Mb locus, using a dedicated single core processor.

Method	Mean run time (minutes)
Fixed-effects meta-analysis (METASOFT)	0.010
Random-effects meta-analysis (METASOFT)	0.010
Meta-regression (MR-MEGA)	0.84
MANTRA	66
PAINTOR	1.2

Table S5. Sample characteristics, imputation and analysis of GWAS contributing to trans-ethnic meta-analysis of eGFR in 71,461 individuals from the COGENT-Kidney Consortium.

Study acronym	Ethnicity	Sex	Sample size	eGFR mean (SD)	Pre-phasing and imputation			Association analysis		
					Software	Quality	SNPs	Software	Covariates	λ_{GC}
PIVUS	European (Sweden)	Males	471	83.8 (19.9)	SHAPEITv2 IMPUTEv2	info≥0.4	9,316,737	SNPTESTv2	Age, sex, 2 PCs	0.982
		Females	473	77.9 (20.2)						
ULSAM	European (Sweden)	Males	1,080	75.2 (11.3)	SHAPEITv2 IMPUTEv2	info≥0.4	9,388,420	SNPTESTv2	Age, 2 PCs	1.013
		Females	0	N/A						
AUSTWIN	European (Australia)	Males	4,662	76.6 (15.8)	MaCH minimac	r^2≥0.3	8,584,822	MERLIN	Age, sex, sub-study, 10 PCs	1.120
		Females	7,096	75.1 (16.5)						
WHI-MS	European (USA)	Males	0	N/A	Beagle minimac	r^2≥0.3	8,814,333	ProbAbel/R	Age, centre, 10 PCs	1.025
		Females	5,655	85.6 (17.8)						
WHI-GARNET	European (USA)	Males	0	N/A	Beagle minimac	r^2≥0.3	8,864,693	ProbAbel/R	Age, centre, 10 PCs	1.018
		Females	4,116	88.1 (19.3)						
BBJ	East Asian (Japan)	Males	12,802	100.2 (28.5)	MaCH minimac	r^2≥0.5	6,581,000	mach2qtl	None	1.058
		Females	10,734	109.1 (31.0)						
HCHS/SOL	Hispanic/Latino (USA)	Males	5,179	95.5 (22.3)	SHAPEITv2 IMPUTEv2	info≥0.4	11,385,919	LMM-OPSa	Age, sex, centre, sampling weights, 5PCs	1.006
		Females	7,420	96.6 (23.4)						
WHI-SHARe	Hispanic/Latino (USA)	Males	0	N/A	MaCH	r^2≥0.3	10,025,812	ProbAbel	Age, centre, 10 PCs	1.027
		Females	3,549	94.7 (21.9)						
	African American (USA)	Males	0	N/A	MaCH	r^2≥0.3	15,345,552	ProbAbel	Age, centre, 10 PCs	1.033
		Females	8,224	80.1 (19.4)						

SD: standard deviation.

Table S6. Sample characteristics of GWAS contributing to trans-ethnic meta-analysis of T2D susceptibility in 22,086 cases and 42,539 controls from the T2D-GENES Consortium.

Study	Ancestry group (country of origin)	Case-control status	Sample characteristics				
			Sample size (males/females)	Age (years) mean (SD)	Age at onset (years) mean (SD)	Fasting glucose (mmol/l) mean (SD)	BMI (kg/m^2) mean (SD)
DGDG	European: French (France)	Cases	679 (413/266)	59.5 (10.1)	45.1 (8.4)	9.2 (3.1)	25.9 (2.8)
		Controls	697 (281/416)	53.9 (5.6)		5.1 (0.4)	23.2 (1.8)
FHS	European (USA)	Cases	677 (386/287)	63.7 (12.4)	N/A	8.6 (2.8)	31.4 (6.5)
		Controls	7,660 (3,441/4,219)	52.3 (16.0)		5.3 (0.5)	27.0 (5.1)
FUSION	European: Finnish (Finland)	Cases	1,160 (653/507)	62.9 (7.6)	53.7 (9.1)	9.4 (3.1)	30.2 (4.7)
		Controls	1,172 (572/600)	63.6 (7.4)		5.3 (0.5)	27.1 (3.9)
LONGENITY	European: Ashkenazim (USA)	Cases	119 (45/74)	89.6 (13.5)	N/A	N/A	N/A
		Controls	465 (147/318)	85.2 (15.2)		N/A	N/A
WTCCC	European: UK (UK)	Cases	1,924 (1,118/806)	58.6 (9.2)	50.3 (9.2)	N/A	30.7 (6.1)
		Controls	2,938 (1,446/1,492)	N/A		N/A	N/A
Starr County	Mexican American (USA)	Cases	837 (333/504)	56.5 (11.8)	46.7 (10.9)	10.0 (4.1)	31.8 (6.4)
		Controls	436 (137/299)	37.6 (9.0)		4.7 (0.5)	29.5 (6.5)
INDICO	South Asian: Indian (North India)	Cases	1,126 (652/474)	53.43(10.67)	46.02(10.39)	8.59(3.36)	25.27(4.21)
		Controls	1,135 (597/538)	52.35(10.13)		4.76(0.66)	24.45(4.78)
LOLIPOP	South Asian: Indian (UK)	Cases	1,783 (1,478/305)	59.4 (9.2)	N/A	8.6 (3.1)	28.1 (4.6)
		Controls	4,773 (4,048/725)	53.9 (10.7)		5.2 (0.6)	26.8 (4.2)
PROMIS	South Asian: Pakistani (Pakistan)	Cases	2,310 (1,765/545)	55.0 (9.3)	N/A	N/A	26.0 (4.0)
		Controls	6,698 (5,561/1,137)	52.9 (10.5)		N/A	25.3 (3.9)
SINDI	South Asian: Indian (Singapore)	Cases	977 (531/446)	60.7 (9.9)	N/A	9.7 (4.4)	27.1 (5.1)
		Controls	1,169 (566/603)	55.7 (9.7)		5.4 (1.1)	25.3 (4.4)
BBJ	East Asian: Japanese (Japan)	Cases	4,470 (3,027/1,429)	65.8 (10.0)	N/A	N/A	25.3 (3.3)
		Controls	3,071 (1,600/1,300)	52.1 (15.0)		N/A	24.1 (3.0)
CAGE	East Asian: Japanese (Japan)	Cases	931 (623/308)	66.1 (9.5)	N/A	N/A	24.4 (3.4)
		Controls	1,404 (844/560)	65.9 (7.4)		N/A	23.1 (3.0)
CLHNS	East Asian: Filipino (Philippines)	Cases	158 (0/158)	49.6 (6.1)	N/A	N/A	24.4 (3.4)
		Controls	1,523 (0/1,523)	48.3 (6.1)		N/A	23.1 (3.0)
Hong Kong	East Asian: Chinese (Hong Kong)	Cases	462 (222/240)	56.9 (13.0)	47.5 (13.9)	9.6 (3.7)	25.8 (5.3)
		Controls	744 (352/392)	37.2 (16.3)		4.7 (0.4)	20.8 (2.0)
KARE	East Asian: Korean (Korea)	Cases	1,042 (539/503)	56.4 (8.6)	N/A	7.0 (2.6)	25.5 (3.3)
		Controls	2,943 (1,355/1,588)	51.5 (8.6)		4.5 (0.4)	24.1 (3.0)
SDCS/SP2(1)	East Asian: Chinese (Singapore)	Cases	1,082 (402/680)	65.1 (9.7)	55.7 (12.0)	N/A	25.3 (3.9)
		Controls	1,006 (217/789)	47.7 (11.1)		4.7 (0.5)	22.3 (3.7)
SDCS/SP2(2)	East Asian: Chinese (Singapore)	Cases	928 (602/326)	63.7 (10.8)	52.2 (14.4)	N/A	25.4 (3.8)
		Controls	939 (599/340)	46.7 (10.2)		4.7 (0.5)	22.8 (3.4)
SiMES	East Asian: Malay (Singapore)	Cases	794 (388/406)	62.3 (9.9)	54.4 (11.2)	N/A	27.8 (4.9)
		Controls	1,240 (595/645)	56.9 (11.4)		N/A	25.1 (4.8)
JHS	African American (USA)	Cases	631 (212/419)	59.4 (10.5)	49.9 (11.7)	7.7 (3.1)	34.2 (7.1)
		Controls	2,526 (980/1,546)	53.6 (13.1)		5.0 (0.5)	31.3 (7.3)

SD: standard deviation
